# *Hemidesmus indicus* and *Hibiscus rosa-sinensis* Affect Ischemia Reperfusion Injury in Isolated Rat Hearts

**DOI:** 10.1155/2011/802937

**Published:** 2010-09-07

**Authors:** Vinoth Kumar Megraj Khandelwal, R. Balaraman, Dezider Pancza, Táňa Ravingerová

**Affiliations:** ^1^Institute for Heart Research, Centre of Excellence for Cardiovascular Research, Slovak Academy of Sciences, 840 05 Bratislava, Slovakia; ^2^Pharmacy Department, Faculty of Technology & Engineering, M. S. University of Baroda, 390 001 Vadodara, Gujarat, India

## Abstract

*Hemidesmus indicus* (L.) R. Br. (HI) and *Hibiscus rosa-sinensis* L. (HRS) are widely used traditional medicine. We investigated cardioprotective effects of these plants applied for 15 min at concentrations of 90, 180, and 360 *μ*g/mL in Langendorff-perfused rat hearts prior to 25-min global ischemia/120-min reperfusion (I/R). Functional recovery (left ventricular developed pressure—LVDP, and rate of development of pressure), reperfusion arrhythmias, and infarct size (TTC staining) served as the endpoints. A transient increase in LVDP (32%–75%) occurred at all concentrations of HI, while coronary flow (CF) was significantly increased after HI 180 and 360. Only a moderate increase in LVDP (21% and 55%) and a tendency to increase CF was observed at HRS 180 and 360. HI and HRS at 180 and 360 significantly improved postischemic recovery of LVDP. Both the drugs dose-dependently reduced the numbers of ectopic beats and duration of ventricular tachycardia. The size of infarction was significantly decreased by HI 360, while HRS significantly reduced the infarct size at all concentrations in a dose-dependent manner. Thus, it can be concluded that HI might cause vasodilation, positive inotropic effect, and cardioprotection, while HRS might cause these effects at higher concentrations. However, further study is needed to elucidate the exact mechanism of their actions.

## 1. Introduction

There is an increasing demand for the herbal drug treatment for various ailments, and many plant drugs from traditional medicine like Ayurveda are being explored globally. 

*Hemidesmus indicus* (L.) R. Br. (Asclepiadaceae; HI) is a twining shrub used as folk medicine and an ingredient in Ayurvedic and Unani preparations. It is known as Indian Sarsaparilla (English), Ananta, Gopasuta, Sariva (Sanskrit), Anantamul (Hindi), Ushba Hindi (Urdu), Ushbahindi (Persian), and Irimusk (Sinhalese) [1]. The root extract of HI was used for preparing herbal soft drinks and as food during famine [2]. The plant has been used traditionally for the treatment of blood disorders, low digestion, anorexia, diarrhea, asthma, fever, cough, itching, and skin diseases including leprosy [1]. Various effects of HI, such as hypoglycemic [3], hypolipidemic [4], antioxidant, antithrombotic [5], antiinflammatory [6], antiulcerogenic [7], hepatoprotective [8], renoprotective [9], and neutralization of viper venom [10] have been reported. It mainly comprises saponins, tannins, hemidesmine, hemidesmol, hemidesterol, stearoptin, pregnane glycosides, *β*-sitosterol, indicusin, coumarin, volatile oils, triterpines, flavonoids, and so forth [1, 7].

*Hibiscus rosa-sinensis* L. (Malvaceae; HRS) is an ornamental plant native to China, and found in India and Philippines. It is called as Chinese rose, Shoe flower (English), Arkapriya, Japapushpa (Sanskrit), Jasund (Hindi), Angharee-hind (Persian), and Wadamal (Sinhalese) [1]. In some regions, the flowers of HRS are eaten raw or cooked [11] and made into a kind of pickle or used as a dye for coloring foods, such as preserved fruits and cooked vegetables [12, 13]. The young leaves are sometimes used as a substitute for spinach [12, 13], while the roots are also edible, but are fibrous, mucilaginous, and without very much flavor [14]. In addition to its traditional value as emollient, demulcent, emmenagogue, antiinflammatory, refrigerant, aphrodisiac, anodyne, and laxative, various researchers had described the use of the flower to treat heart disorders [1, 10, 15]. Sachdewa and Khemani [16] demonstrated the antidiabetic activity of HRS in diabetic rats and the effect was comparable with glibenclamide. It has been also shown to be beneficial in fever and bronchial catarrh [16]. It is known to possess various activities like antidiarrheal, antiphologistic, antispermatogenic, androgenic, antitumor [16], antiestrogenic [17], antiimplantation [18, 19], wound Healing [20], anticonvulsant [21], and so forth. It mainly consists of flavonoids, anthocyanins, quercetin, cyanidin, kaempferol, hydrocitric acid, and so forth [1, 22].

However, till date, no research work has been performed to study the effects of HI and HRS in isolated heart preparation. Hence, this study was initiated to evaluate the potential myocardial protective effect of both the drugs in the model of ischemia-reperfusion (I/R) injury in rat hearts *in vitro*.

## 2. Materials and Methods

### 2.1. Preparation of the Extract

Standardized dry extracts of HI and HRS (prepared as below) were kindly gifted by Rumi herbal research institute, Chennai, India. In brief, dried roots of HI were coarsely powdered and refluxed with 50% ethanol by hot percolation method and extracted. The yield was 23.18% of black-brown extractives containing 2.87 mg % of saponins and 1.62 mg % tannins, heavy metals—arsenic—not more than (NMT) 1 parts per million (ppm), lead NMT 1 ppm, and *E. coli* and *Salmonella* were absent. Dried flowers of HRS were coarsely powdered and refluxed with 80% ethanol by hot percolation method and extracted. It yielded 15.6% of dark-brown extractives containing 4.16 mg % hydrocitric acid, heavy metals—arsenic—NMT 1 ppm, lead NMT 1 ppm, and *E. coli* and *Salmonella* were absent.

The dry extract (600 mg) was added to 15 mL of boiling distilled water and boiled for 2 min. The decoction was cooled and centrifuged to separate any undissolved material and the supernatant was considered as a stock solution containing 40 mg/mL of the extract [23]. The decoction was prepared fresh every day.

### 2.2. Animals

Male adult Wistar rats (230–270 g) were used in this study. Rats were housed under standard conditions and supplied with drinking water and food *ad libitum*. All procedures and experimental protocols were performed in accordance with the *Guide for the Care and Use of Laboratory Animals* published by the US National Institutes of Health and approved by the Animal Health and Animal Welfare Division of the State veterinary and Food Administration of the Slovak Republic.

### 2.3. Perfusion Technique

The rats were anesthetized (sodium pentobarbitone, 60 mg/kg, i.p.) and heparinized (500 IU, i.p.) [24]. Hearts were excised and rapidly mounted on the Langendorff perfusion apparatus. Retrograde perfusion in a nonrecirculating mode was established at a constant perfusion pressure of 70 mmHg and 37°C. Krebs-Henseleit buffer (KHB) gassed with 95% O_2_ and 5% CO_2_ (pH 7.4) containing (mM) NaCl 118.0, KCl 4.7, MgSO_4_·7H_2_O 1.18, NaHCO_3_ 25.0, KH_2_PO_4_ 1.18, CaCl_2_·2H_2_O 2.25, and glucose 11.1 was used as the perfusion medium. The perfusate was filtered through a 5-*μ*m porosity filter (Millipore), before it entered the heart. An epicardial electrogram was registered using two stainless steel electrodes, one attached to the apex of the heart and the other to the aortic cannula.

Left ventricular (LV) pressure was measured using a nonelastic water-filled balloon inserted into the left ventricle via the left atrium (adjusted to obtain end-diastolic pressure of 4–7 mmHg) and connected to a pressure transducer (MLP844 Physiological Pressure Transducer, ADInstruments). Left ventricular developed pressure (LVDP, systolic minus diastolic pressure), maximal rate of pressure development (+dP/dt_max _) as an index of contraction, heart rate (HR; derived from electrogram), and coronary flow (CF) were monitored continuously. The hearts were allowed to stabilize (15 min) before further interventions. Baseline values of functional parameters were recorded after stabilization and recording of the data was performed until the end of an experiment, except for the contractile function, as the balloon was deflated after 40 min of R. Heart function and arrhythmias were analyzed using PowerLab/8SP Chart 5 software (ADInstruments). 

Recovery of function was expressed as a percentage of preischemic baseline values.

### 2.4. Experimental Protocol

The experimental protocol consisted of a stabilization period (15 min), perfusion with drugs dispersed in KHB at the required concentrations for 15 min, global ischemia (25 min), and reperfusion period (120 min). 

All animals were randomly divided to the following groups (seven rats per group).

1. Control (C): hearts were perfused with KHB throughout the experiment.
2. HI 90: hearts were perfused with HI extract at a concentration of 90 *μ*g/mL in KHB for 15 min, prior to ischemia and reperfusion with KHB.
3. HI 180: hearts were perfused with HI extract at a concentration of 180 *μ*g/mL in KHB for 15 min, prior to ischemia and reperfusion with KHB.
4. HI 360: hearts were perfused with HI extract at a concentration of 360 *μ*g/mL in KHB for 15 min, prior to ischemia and reperfusion with KHB.
5. HRS 90: hearts were perfused with HRS extract at a concentration of 90 *μ*g/mL in KHB for 15 min, prior to ischemia and reperfusion with KHB.
6. HRS 180: hearts were perfused with HRS extract at a concentration of 180 *μ*g/mL in KHB for 15 min, prior to ischemia and reperfusion with KHB.
7. HRS 360: hearts were perfused with HRS extract at a concentration of 360 *μ*g/mL in KHB for 15 min, prior to ischemia and reperfusion with KHB.

### 2.5. Quantification of Arrhythmias

Susceptibility to ventricular arrhythmias was analyzed from the electrogram recording during the first 10 min of R, as per the guidelines for the study of ischemia- and reperfusion-induced arrhythmias, known as the Lambeth conventions [25]. We focused on the measurement of the total number of ventricular premature beats (VPB), as well as on the total duration of the episodes of ventricular tachycardia (VT), which was defined as a run of four or more consecutive ectopic beats.

### 2.6. Infarct Size Determination

The measurement of infarct size using triphenyl tetrazolium staining was essentially identical to that described by Ravingerová et al. [26]. In brief, at the end of R, the hearts were stained with 1% 2,3,5-triphenyl tetrazolium chloride (Sigma, USA) dissolved in 0.1 M phosphate buffer (pH 7.4). The hearts were then stored overnight in 10% neutral formaldehyde solution and cut perpendicularly to the long axis of the ventricle into 1-mm thick slices. The infarct area (IA) and the area at risk (AR), which in the setting of global ischemia was the whole mass of the left ventricle, were measured by a computerized planimetric method. The infarct size was normalized to the size of the area at risk (IA/AR). 

### 2.7. Statistical Evaluation

The data were expressed as mean ± S.E.M. The statistical analysis was performed with one-way ANOVA followed by Newman-Keuls multiple comparison test or two-way ANOVA followed by Bonferroni post tests. Differences were considered significant when *P* ≤ .05.

## 3. Results

### 3.1. Preischemia

After 15-min stabilization of all the hearts with KHB, the perfusion was switched to the drug-containing KHB solution. During perfusion with HI, we observed a transient increase in LVDP that occurred during perfusion with HI 90 (32%), HI 180 (52%), and HI 360 (75%) when compared with predrug values.  Table 1 shows that at the end of 15 min perfusion with HI, LVDP and HR were similar to those of pre-drug values at all doses, while CF was significantly increased by HI 180 (*P* < .05) and HI 360 (*P* < .01) when compared with pre-drug values (8.8 ± 0.3 mL/min).

When the hearts were perfused with HRS, a transient increase in LVDP at HRS 180 (21%) and HRS 360 (55%) was observed when compared with pre-drug values. At the end of 15-min perfusion with HRS, there was only a tendency to increase CF, and no significant changes in LVDP and HR at all doses (Table 1).

### 3.2. Post-Ischemic Recovery of Function

#### 3.2.1. LVDP

**Figure 1:**
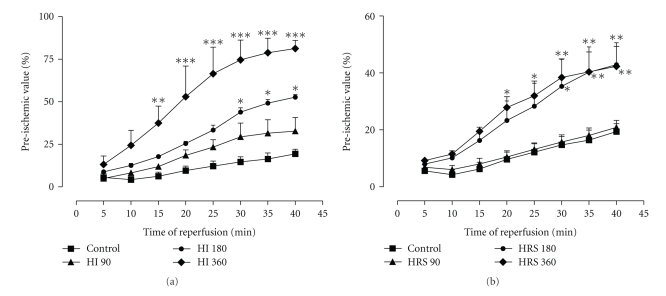
Effect of HI (a) and HRS (b) on the time course of LVDP recovery after I/R expressed as a percentage of the baseline values. *P < .05, **P < .01, ***P < .001 versus C. *n* = 7.

Figure 1(a) shows the time course of post-ischemic recovery of LVDP. At 40 min of R, HI 180 and HI 360 significantly (*P* < .05 and *P* < .001, resp.) improved the recovery of LVDP to 52.7 and 81.2%, respectively, when compared with 19.4% in the nontreated C group. The changes in the LVDP recovery induced by HI 90 were not significant at any time point.

Figure 1(b) depicts the time course of post-ischemic LVDP recovery in the presence of HRS. HRS 90 did not exert any effect when compared with C. However, HRS 180 exerted a more pronounced effect on recovery of LVDP and was significant at few time points. HRS 360 showed a significant recovery at all time points after 20 min of R. At the end of 40 min of I/R, both HRS 180 and HRS 360 induced almost similar recovery which was significantly (*P* < .01) better when compared with C.

#### 3.2.2. +dP/dt

**Figure 2:**
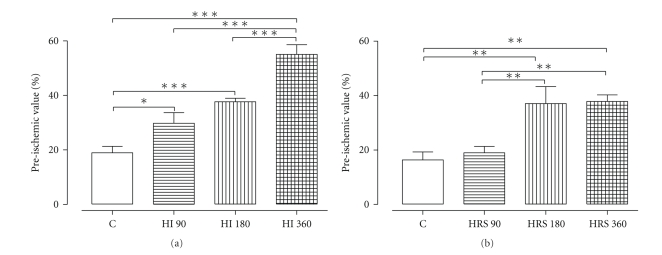
Effect of HI (a) and HRS (b) on +dP/dt_max_ at 40 min of R. *P < .05, **P < .01, ***P < .001, *n* = 7.

Figure 2(a) shows the recovery of +dP/dt_max_ at 40 min of I/R. When compared with C, the recovery was significant when the hearts were perfused with HI 90 (*P* < .05), HI 180 (*P* < .001), and HI 360 (*P* < .001). The recovery of +dP/dt_max_ was dose-dependent, that is, HI 360 induced a significantly higher recovery (*P* < .001) than HI 90 and HI 180.

The recovery of +dP/dt_max_ was significantly (*P* < .01) better when compared with C, when the hearts were perfused with HRS 180 and HRS 360. HRS 180 and HRS 360 showed a significantly stronger effect (*P* < .01) than HRS 90 (Figure 2(b)).

#### 3.2.3. LVEDP

**Figure 3:**
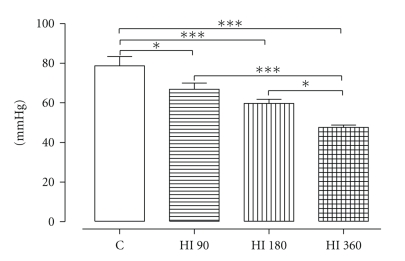
Effect of HI on LVEDP at 40 min of R. *P < .05, ***P < .001, *n* = 7.

Figure 3 shows the recovery of LVEDP, which was significantly lowered by all concentrations of HI, HI 90 (*P* < .05), HI 180 (*P* < .001), and HI 360 (*P* < .001). Furthermore, perfusion with HI 360 led to a significantly better recovery of LVEDP when compared with HI 90 and HI 180 (*P* < .001 and *P* < .05, resp.).

HRS did not cause an improvement of LVEDP recovery (in mmHg) at any concentration HI 90 (77.8 ± 3.5), HI 180 (68.2 ± 1.8), HI 360 (67.3 ± 2.4), in comparison to C (78.6 ± 4.7).

#### 3.2.4. Arrhythmias

**Figure 4:**
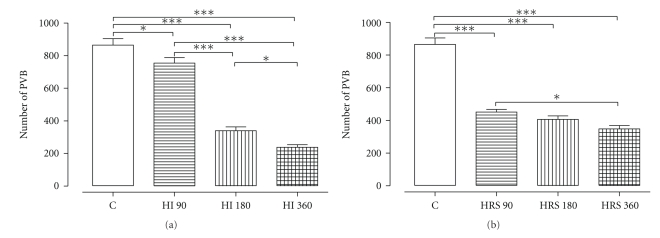
Effect of HI (a) and HRS (b) on arrhythmias (PVB) during the first 10 min of R. *P < .05, ***P < .001, *n* = 7.

HI exerted a significant antiarrhythmic protection at HI 90, HI 180, and HI 360 manifested by a reduced number of PVB (*P* < .05, *P* < .001, and *P* < .001, resp.). The protection was also dose dependent (Figure 4(a)), as perfusion with HI 360 resulted in a significantly lower number of PVB than HI 90 (*P* < .001) and HI 180 (*P* < .05), while HI 180 induced a significantly (*P* < .001) lower number of PVB than HI 90. There was also a significantly (*P* < .01) shorter duration of episodes of VT at HI 180 (9.8 ± 3.0 s) and HI 360 (5.3 ± 2.8 s), but not at HI 90 (28.5 ± 9.7 s), when compared with 39 ± 6.5 s in C.

Interestingly, HRS 90, HRS 180, and HRS 360 significantly (*P* < .001) reduced the number of PVB and decreased the duration of VT (*P* < .05) to 19.8 ± 6.8, 13.7 ± 5.6, and 13.2 ± 5.1 s, respectively, when compared with nontreated C (Figure 4(b)).

#### 3.2.5. Infarct Size

The size of infarction (percentage of the risk area; IA/AR) was significantly reduced only after administration of HI 360 (20.3 ± 1.4%; *P* < .01) and not at HI 90 (33.5 ± 5.3%) and HI 180 (30.1 ± 4.9%), when compared with C (43.2 ± 2.4%).

The infarct size was significantly (*P* < .01) smaller at HRS 90 (29.4 ± 4.7%), HRS 180 (24.8 ± 3.6%), and HRS 360 (22.5 ± 2.4%), when compared with C.

## 4. Discussion

The extracts of HI and HRS were tested for their potential protective effect on I/R-induced lethal injury and functional deterioration. The effects of the extracts were evaluated before I and during R. The widely used model of 25-min global I for optimum functional deterioration [27], followed by 120 min of R for sufficient development of necrosis and infarct size determination in the Langendorff setup [28–30] was utilized. 

HI had a dose-dependent effect on the recovery of LVDP and +dP/dt_max _. HRS had a similar effect on the recovery of LVDP and +dP/dt_max_ at HI 180 as in HRS 360 and no protection was observed at HRS 90, suggesting that HRS 180 is the minimum dose required to increase the recovery of contractile function. The significantly better recovery of LVEDP and attenuation of post-ischemic diastolic dysfunction at all three doses of HI infers that HI could improve myocardial relaxation and may reduce the edema caused by I/R injury [31]. In contrast, HRS did not have any significant activity towards the relaxation of the cardiac muscle at any dose.

HI could protect the heart from arrhythmias at all doses in a dose-dependent manner manifested by a reduced number of PVB (extra heart beat caused by abnormal electrical activity). In addition, a significantly lower total duration of episodes of VT (rapid heart rhythm) was observed at higher doses. Interestingly, HRS at all doses had a significant protection against arrhythmias. The size of infarction (death of a macroscopic area of cardiac tissue) was significantly reduced by HI at the highest dose, while HRS significantly lowered the infarct size at all the doses. In comparison, after 25-min I, HI exerted a higher protective activity against functional deterioration and a moderate protection against arrhythmias and infarct size, while HRS had a moderate effect on functional recovery and a stronger protection in terms of antiarrhythmic effect and infarct size limitation.

In two different studies recently performed in our laboratory in a similar model, N-acetylcysteine (4 mM) [27] and quercetin (15 *μ*M) [32] were found to protect the myocardium against I/R. In the present study, the recovery of various parameters in the presence of both the extracts was comparable with that of N-acetylcysteine (LVDP—50%) and quercetin (LVDP—39.4%, +dP/dt_max _—30.9%, IA/AR—14.3%). At higher concentrations, the extracts showed a better effect than quercetin [27, 32]. Functional deterioration and severe arrhythmias upon reperfusion were found to be related, to a certain extent, to an excessive generation of reactive oxygen species (ROS) during prolonged I/R [33–36]. ROS may also participate in I/R injury through the depression of sarcoplasmic reticulum (SR) Ca^2+^ handling by modulating gene expression in the I/R heart [37]. This has been verified by the efficacy of antioxidants and scavengers in the experimental settings of acute I/R [38–41]. Similarly, pre-treatment with antioxidants, such as melatonin [38, 39] and N-acetylcysteine [27] prior to I reduced the severity and duration of R-induced ventricular arrhythmias in isolated perfused rat hearts, attenuated calcium overload of the heart [40, 42], and improved post-ischemic recovery of the contractile function [38].

**Figure 5:**
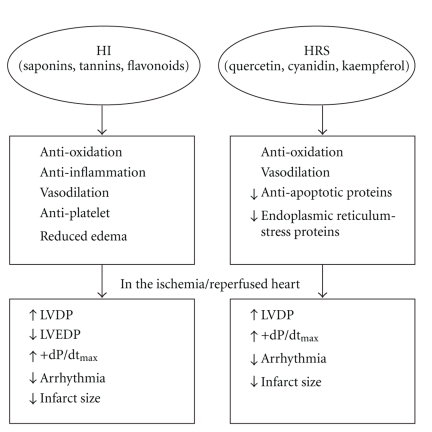
Potential mechanisms for the beneficial effects of HI and HRS in I/R rat hearts.

The previously reported antioxidant effect of HI [5, 43] may be associated with tannins, one of the main constituents [44]. Likewise, saponins have also been shown to have beneficial effects on cardiovascular diseases [45]. Flavonoids produce vasodilation by regulating endothelial nitric oxide (NO) production [46] and interaction with ion channels [47]. Moreover, flavonoids are known to protect the I/R-induced myocardial injury by their multifaceted properties, such as antioxidant, antiinflammatory, vasodilatory, and antiplatelet aggregation [47]. Therefore, it is conceivable that the cardioprotective effect of HI may be related to the combined effects of saponins, tannins, and flavonoids. HRS has been shown to enhance the endogenous antioxidant activity and protect the heart from isoproterenol-induced injury [48]. Quercetin has been shown to reduce blood pressure and exhibit endothelium-dependent vasodilation by enhancing eNOS activity [46, 49–51]. In addition, cyanidin and quercetin are known to possess antioxidant activity [48, 49]. Kim et al. [52] have shown that kaempferol protected the cardiac muscle cells against I/R-induced damage by increasing the expression of antiapoptotic protein and downregulating the expression of endoplasmic reticulum stress proteins. Thus, the combined effect of constituents of HRS, such as quercetin, cyanidin, and kaempferol might be responsible for the beneficial effects (Figure 5). 

In conclusion, HI might cause vasodilation, positive inotropic effect, and cardioprotection, while HRS might cause these effects at higher concentrations. In addition, based on the drug effects observed at lower doses, it could be suggested that the suppression of arrhythmias results in a smaller size of infarction than that achieved by the protection against contractile dysfunction. However, further study is required to explore the *in vivo* activity of both the plants.

## Figures and Tables

**Table 1:** Effect of 15-min perfusion with HI and HRS on hemodynamic parameters of the isolated rat heart. LVDP: left ventricular developed pressure (LV systolic minus diastolic pressure); HR: heart rate; CF: coronary flow; BD: Before Drug (pre-drug values). *P < .05, **P < .01 versus BD (baseline).

	BD	HI			BD	HRS		
		pre-ischemia				pre-ischemia		
		HI 90	HI 180	HI 360		HRS 90	HRS 180	HRS 360
LVDP (mmHg)	89.5 ± 9.1	83.9 ± 2.2	84.8 ± 1.9	82.2 ± 7.5	83.9 ± 4.0	77.6 ± 3.5	82.6 ± 3.2	80.7 ± 2.8
HR (BPM)	302.6 ± 22.6	290.1 ± 31.5	286.6 ± 9.7	299.4 ± 33.1	305.8 ± 11.4	284.1 ± 9.3	290.0 ± 23.8	292.1 ± 13.8
CF (mL/min)	8.8 ± 0.3	14.1 ± 2.8	17.7 ± 2.5*	20.6 ± 2.7**	8.8 ± 0.4	10.5 ± 0.9	11.0 ± 2.2	12.4 ± 1.6
